# Reconsideration of Dynamic Force Spectroscopy Analysis of Streptavidin-Biotin Interactions

**DOI:** 10.3390/ijms11052134

**Published:** 2010-05-13

**Authors:** Atsushi Taninaka, Osamu Takeuchi, Hidemi Shigekawa

**Affiliations:** Institute of Applied Physics, CREST-JST, University of Tsukuba/1-1-1 Tennodai, Tsukuba 305-8573, Japan; E-Mails: jun_t@bk.tsukuba.ac.jp (A.T.); osamu@big.or.jp (O.T.)

**Keywords:** dynamic force spectroscopy, atomic force microscopy, site-selective analysis, streptavidin-biotin interaction

## Abstract

To understand and design molecular functions on the basis of molecular recognition processes, the microscopic probing of the energy landscapes of individual interactions in a molecular complex and their dependence on the surrounding conditions is of great importance. Dynamic force spectroscopy (DFS) is a technique that enables us to study the interaction between molecules at the single-molecule level. However, the obtained results differ among previous studies, which is considered to be caused by the differences in the measurement conditions. We have developed an atomic force microscopy technique that enables the precise analysis of molecular interactions on the basis of DFS. After verifying the performance of this technique, we carried out measurements to determine the landscapes of streptavidin-biotin interactions. The obtained results showed good agreement with theoretical predictions. Lifetimes were also well analyzed. Using a combination of cross-linkers and the atomic force microscope that we developed, site-selective measurement was carried out, and the steps involved in bonding due to microscopic interactions are discussed using the results obtained by site-selective analysis.

## 1. Introduction

The variety and selectivity of interactions between a pair of functional molecules, for example, DNA, ligand-receptor and antigen-antibody systems, play essential roles in biological processes and molecular devices based on molecular recognition properties [1–6]. However, such interactions include various effects, for example, those of solvent parameters and the manifold structures of functional molecules, which prevent the design of detailed functions in predetermined structures [1–6]. Despite their importance, local variations between two molecules during their chemical reaction may not have been probed by a thermodynamic method and remain hidden behind the averaged function. Therefore, the microscopic probing of the energy landscapes of individual interactions in a molecular complex and their dependence on the surrounding conditions is strongly desired to achieve further advances in biophysics and chemistry and their applications.

Dynamic force spectroscopy (DFS) is a technique that enables us to study the interaction between molecules at the single-molecule level [7–26]. In DFS, the unbinding force applied to a pair of molecules is increased at a constant rate, and the force required to rupture the bond is measured. By analyzing the relationship between the modal rupture force and the logarithm of the loading rate, microscopic potential barrier landscapes (the distance from the potential minimum) and the lifetimes of bonds can be obtained [7–26]. However, the obtained results for potential barriers and bond lifetimes differed among the previous studies [9–13], and some results were inconsistent with theoretical predictions. Table 1 shows previously obtained results for the interactions of streptavidin/biotin complexes.

The different measurement conditions, such as the types of cross-linkers, substrates and measurement systems shown in Table 1, may affect the results. For example, streptavidin is directly fixed to a cantilever in the case of [13]. The length of the molecular chains used for linkers is not the same for each method as noted in Table 1. However, in a conventional DFS analysis, since the sampling rate is not sufficiently high at a high loading rate and it is difficult to maintain a constant loading rate at a low loading rate, even though the same measurement conditions are used it is difficult to perform measurements to acquire accurate rupture forces [27], resulting in the difference in the slope of the relationship between the modal rupture force and the logarithm of the loading rate, which provides potential barrier positions and lifetimes. In addition, the existence of the cross-linker molecule used to fix the target molecules to the cantilever and substrate affects force measurement [19–24]. These points must be considered before discussing the details of molecular interactions.

We have developed an atomic force microscopy (AFM) technique that enables the precise analysis of molecular interactions on the basis of DFS [15,16]. In this paper, after examining the performance of this spectroscopy technique in detail, we discuss the results obtained for determining the landscapes of streptavidin-biotin interactions. The results obtained by this spectroscopy technique showed good agreement with theoretical predictions. Furthermore, using the combination of cross-linkers and the atomic force microscope that we developed, site-selective measurement was carried out. The steps involved in bonding due to microscopic interactions will be discussed using the results obtained by site-selective analysis.

## 2. Developed Techniques and Experimental Methods

First, we explain the methodology we developed and the details of sample preparation.

### 2.1. For Precise and Deeper Analysis

In DFS, the unbinding force applied to a pair of molecules is increased at a constant rate, and the force required to rupture the bond is measured. The lifetime of a molecular bond depends on the potential barrier height. When a tensile force is applied to a bond, the potential landscape is deformed; thus, the barrier height decreases. When the tensile force is applied at a constant loading rate, the probability distribution of the rupture force can be expressed as

(1) $$P_{\left(f\right)}=C\;\text{exp}\left\{\overset{(f-f^{*})x_{b}}{\;}/\underset{k_{B}T}{\;}\right\}\text{exp}\left[1-\text{exp}\left\{\overset{(f-f^{*})x_{b}}{\;}/\underset{k_{B}T}{\;}\right\}\right]$$

where *P*_(*f*)_, *f*, f*, *x*_b_, *k*_B_, *T*, and *C* are the probability distribution of the rupture force, the rupture force, the modal rupture force, the distance of the potential barrier position from the potential bottom, the Boltzmann constant, the temperature and a constant, respectively [9,10,15,16]. According to Equation 1,f* linearly depends on the logarithm of the loading rate *r*_0_ (d*f*/d*t*), as shown by

(2) $$f^{*}=\overset{k_{B}T}{\;}/\underset{x_{b}}{\;}\left\{\text{ln}\;r_{0}+\text{ln}\left(\overset{t_{\text{off}}(0)x_{b}}{\;}/\underset{k_{B}T}{\;}\right)\right\}$$

where *t*_off_(0) is the lifetime of the molecular bond. Equation 2 indicates that the potential barrier position can be obtained from the slope of the linear relationship. When using Equations 1 or 2, it is essential to maintain a constant loading rate [15–20]. However, when the DFS measurement is carried out by AFM with a cross-linker molecule, a constant retraction velocity does not result in a constant loading rate because of the stretching of the cross-linker molecule [19,20]. Therefore, we introduce a mechanism for controlling the applied force to maintain a constant loading rate. To satisfy this requirement, we have developed an AFM system with a force feedback system that enables precise analysis by DFS [15–18], which features (1) fine control of the loading rate to reduce the effect of the soft cross-linker connecting a sample molecule to the tip or substrate [15–18] and (2) a high sampling rate (100 kHz) to obtain a sufficient amount of data at a high loading rate [15,16]. A high sampling rate (at least 1 kHz) is required to resolve the subtle processes underlying the interactions between the pair of molecules [27]. Furthermore, since AFM measurement is stable under various pHs, different buffer solutions with different pHs can be used.

### 2.2. Structure of Streptavidin Molecule

**Figure 1.**
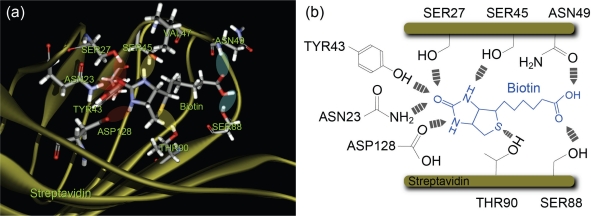
Structure of streptavidin-biotin complex (reproduced from [28,29]).

We used the streptavidin-biotin complex, a typical ligand-receptor system, as a sample [28,29]. Streptavidin is a tetrameric protein that has a high affinity for biotin molecules. Each monomer of streptavidin bonds with one biotin molecule. The binding pocket of streptavidin has several reaction sites with a hydrogen-bonding network, which can be classified into the following three groups depending on the distance from the bottom of the binding pocket (Figure 1) [28,29]: (1) the inner binding sites of amino acid residues SER27, ASN23, TYR43 and ASP128, (2) the middle binding sites SER45 and THR90 and (3) the outer bonding sites ASN49 and SER88. Since streptavidin has a complex structure when considering its chemical reactions, various processes are expected to occur in this system depending on the operating conditions used. Therefore, probing to obtain a deeper understanding of the energy landscapes of individual interaction sites of this system will provide a foundation for designing and controlling the mechanism of chemical reactions between two more complex functional molecules.

### 2.3. Cantilever and Sample Preparation

**Figure 2.**
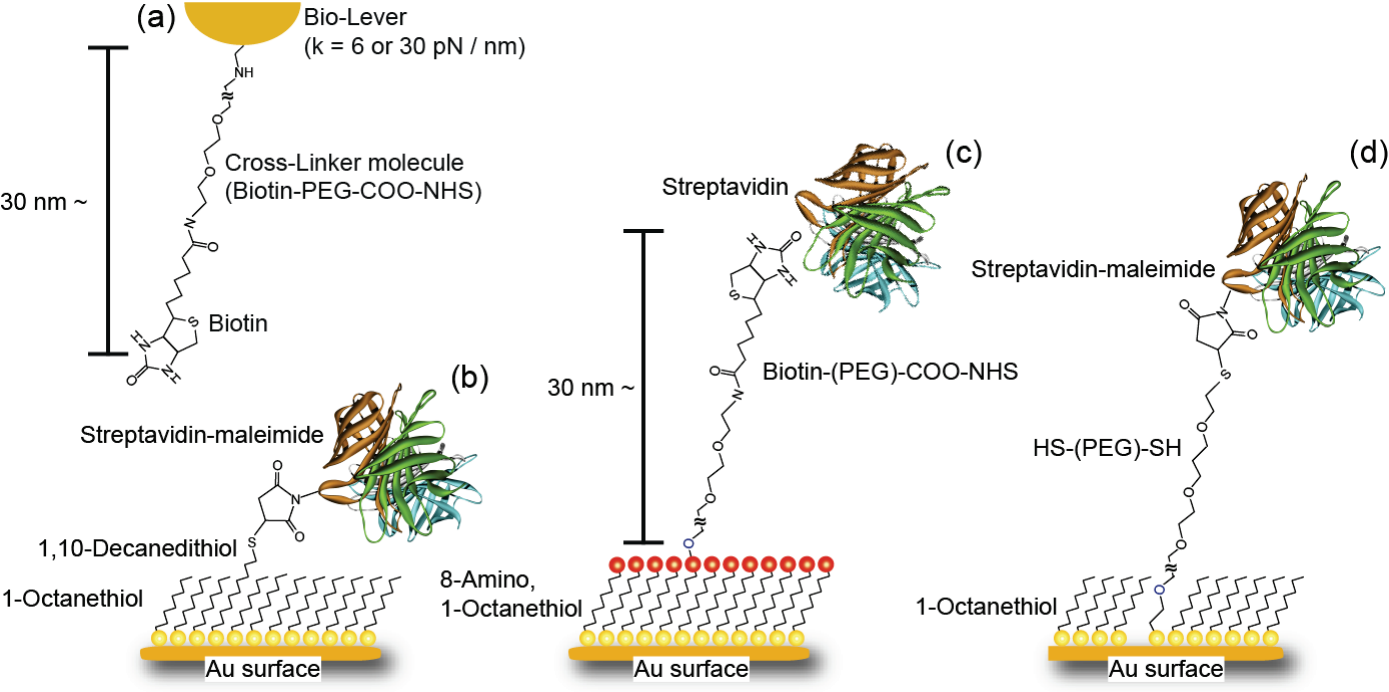
Schematic illustrations of the apex of a modified cantilever (a) and the three types of cross-linker for site-selective analysis; streptavidin is fixed to a SAM of (b) 1,10-decanedithiol/1-octanethiol (1/100 ratio) solution on a Au-coated substrate via a streptavidin-maleimide structure (SM condition), (c) 8-amino, 1-octanethiol molecule on a Au-coated substrate via a biotin-PEG-COO-NHS molecule (B-PEG condition) and (d) HS-(PEG)-SH/1-octanethiol (1/100 ratio) mixed solution on a Au-coated substrate via a streptavidin-maleimide structure (SM-PEG condition).

A rectangular gold-coated cantilever (Bio-Lever, Olympus, 6 pN/nm and 30 pN/nm) was immersed in a solution of 8-amino, 1-octanethiol hydrochloride molecules (Sigma-Aldrich, 1 mM in ethanol) for 48 h to form a closely packed SAM with amino groups on the surface. After rinsing with ethanol, the cantilever was immersed in a solution of biotin-PEG3400-COO-NHS (Shearwater Polymers, 0.1 mM in ethanol) for 20 h to fix a biotin (biotin-PEG) molecule onto the probe apex (Figure 2(a)). Finally, the cantilever was rinsed with ethanol. The formation of closely packed SAMs on the probe apex and substrate was necessary to decrease the strong interaction between the gold layers on both surfaces.

For the microscopic analysis of the energy landscapes and lifetimes of streptavidin-biotin interactions, which will be discussed in Sections 3–3 and 3–4 in detail, we prepared three types of cross-linker, as schematically illustrated in Figure 2. Namely, streptavidin was fixed to a SAM of (b) 1,10-decanedithiol/1-octanethiol (1/100 ratio) mixed solution on a Au-coated substrate via a streptavidin-maleimide structure (SM), (c) an 8-amino, 1-octanethiol molecule on an Au-coated substrate via a biotin-PEG-COO-NHS molecule (B-PEG) and (d) HS-(PEG)-SH/1-octanethiol (1/100 ratio) mixed solution on a Au-coated substrate via a streptavidin-maleimide structure (SM-PEG). To avoid multiple-bonding events, the density of target molecules in the SAM was reduced by adjusting the ratios of 1,10-decanedithiol/1-octanethiol and HS-(PEG)-SH/1-octanethiol molecules to 1/100. The distances between the streptavidin molecule and the substrate are ∼30 nm for the B-PEG and SM-PEG conditions, and ∼1.5 nm for the SM condition, as shown in Figure 2.

**Figure 3.**
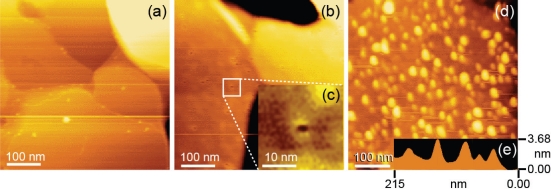
Tapping-mode AFM images of a sample substrate: (**a**) bare Au, (**b**) with 1,10-decanedithiol/1-octanethiol SAM, (**c**) magnified image of an etch pit in (b), (**d**) substrate immersed in 10 mg/l streptavidin-maleimide solution and (**e**) line profile of streptavidin-maleimide molecules. The spring constant of the cantilever used for all measurements was 2 N/m.

First, a thin gold film (100 nm) was evaporated as a substrate onto a freshly cleaved mica surface in a high vacuum at 400 °C and flame-annealed using a hydrogen gas burner for 30 s. A flat Au surface was obtained, as shown by the AFM image in Figure 3(a). Then, for the SM condition, the substrate was immersed in a solution of 1,10-decanedithiol/1-octanethiol (1/100 ratio) (Sigma-Aldrich, 1 mM in ethanol) for 48 h to form a closely packed SAM with thiol groups on the surface. The formation of a flat closely packed SAM with etch pits was confirmed by AFM, as shown in Figure 3(b),(c). After rinsing with ethanol, the substrate was immersed in a solution of streptavidin-maleimide (Sigma-Aldrich, 10 mg/l in phosphate-buffered saline (PBS), pH 7.4: *N*,*N*-dimethylformamide = 99:1) for 5 h to fix streptavidin molecules to the substrate. Finally, the substrate was rinsed with PBS (Figure 2(b)). As shown in Figure 3(d),(e), isolated streptavidin molecules of 2.3–4.4 nm height were observed by AFM. The density of streptavidin molecules is ∼5/100 × 100 nm^2^, which is sufficiently low to enable the measurement of a single bond.

For the B-PEG condition, the substrate was immersed in a solution of 8-amino, 1-octanethiol (Sigma-Aldrich, 1 mM in ethanol) for 48 h to form a closely packed SAM with amino groups on the surface. After rinsing with ethanol, the substrate was immersed in a solution of biotin-PEG-COO-NHS (0.1 mM in ethanol) for 20 h to fix biotin onto the substrate. Finally, the substrate was rinsed with ethanol (Figure 2(c)).

For the SM-PEG condition, the substrate was immersed in a solution of HS-(PEG)-SH/1-octanethiol (1/100 ratio) (SUNBRIGHT, NOF Corporation, 1 mM in ethanol) for 48 h to form a closely packed SAM with thiol groups on the surface. After rinsing with ethanol, the substrate was immersed in a solution of streptavidin-maleimide (Sigma-Aldrich, 10 mg/l in phosphate-PBS, pH 7.4: *N*,*N*-dimethylformamide = 99:1) for 5 h to fix streptavidin molecules to the substrate. Finally, the substrate was rinsed with PBS (Figure 2(d)).

### 2.4. Measurement Procedures

In DFS, the unbinding force applied to a pair of molecules is increased at a constant rate, and the force required to rupture the bond is measured [15–20]. In our experiment, the loading rate was controlled between 10 and 10^5^ pN/s, and 5,000–10,000 approach and retract cycles were carried out to form a histogram for each loading rate measurement. To avoid multiple-bonding events, as mentioned above, the density of target molecules in the SAM was reduced so that the probability of bonding became 5–10% for each tip-sample approach. Furthermore, only single ruptures were counted to remove the errors caused by the effects of multiple-rupture events on the analysis results. All DFS measurements were performed at room temperature.

**Figure 4.**
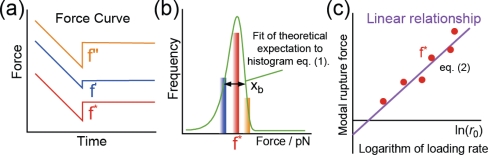
Schematic diagram representing the method of DFS analysis.

In general, for a selected loading rate, many rupture forces are measured from force curves (Figure 4(a)). The measured rupture forces are shown in a histogram, from which the modal rupture force is obtained for each loading rate measurement (Figure 4(b)). In the analysis, the modal rupture forces are plotted as a function of the logarithm of the loading rate (Figure 4(c)), thereby, information concerning the energy landscape of the interaction is derived from the relationship between the modal rupture force obtained from the histogram and the loading rate of the unbinding force. In addition, the width of the histogram also provides the potential barrier position by analysis using Equation 1 (Figure 4(b)) [15,16], which can be used to examine the validity of the results obtained from the slope shown in Figure 4(c).

## 3. Results and Discussion

### 3.1. Confirmation of Measurement Conditions

We first confirmed that no rupture event occurred when streptavidin molecules were not prepared on the substrate. This is because we covered the gold surfaces of the probe and substrate with closely packed SAMs of spacer molecules to prevent direct bonding between their bare surfaces.

**Figure 5.**
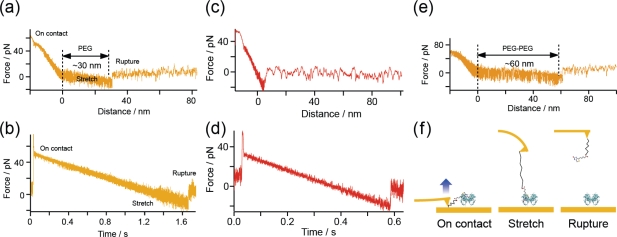
(**a**) and (**b**): Two different forms of the typical force curve obtained using our system [15,16] as functions of (a) distance and (b) time. (**c**) and (**d**) show the data obtained for a direct interaction between the cantilever and the substrate, where no stretching of the PEG molecule is observed. (**e**) shows a typical force curve obtained for the B-PEG condition. (**f**) Schematic diagram of the approach and retract cycle.

Secondly, the stretching motion of a PEG molecule was observed for each rupture event. Figure 5(a),(b) show two different forms of the typical force curve obtained at a loading rate of 39 pN/s under the SM condition (Figure 2(b)) as functions of (a) distance and (b) time. The distance represents the length of the cantilever retraction measured from the zero-force position. Since the cantilever was first moved inward until the force reached 50 pN, a region of negative distance exists. Although the force does not exhibit a linear change in (a) owing to the existence of a PEG molecule, a linear change is shown in (b). This result indicates that a constant loading rate is achieved during the stretching of the PEG molecule. The gradient of the force-distance curve in Figure 5(a) after reaching the zero-force position is smaller than that in the negative-distance region because of the existence of the PEG molecule. The rupture force is 11.5 pN, which is the value expected for the measurement at the loading rate.

When there is a direct interaction between the cantilever and the substrate, no stretching of the PEG molecule is observed and the rupture force should be much larger than 11.5 pN (Figure 5(c),(d)). The distance between the zero-force position and the rupture point includes the amount of bending of the cantilever, which is obtained by dividing the rupture force by the spring constant of the cantilever (6 pN/nm). Therefore, the actual length of the PEG molecule can be estimated by subtracting the amount of bending of the cantilever from the distance of 30.4 nm between the zero-force and rupture positions obtained from Figure 5(a). The obtained length is 28 nm, which is in good agreement with the length expected from the PEG molecules that we used (molecular weight, ∼3400: length, ∼30 nm). Figure 5(e) shows a typical force curve obtained for the B-PEG condition (Figure 2(c)). The length of the PEG molecule, obtained from the PEG-PEG distance (61.4 nm), the rupture force (18.9 pN) and the spring constant of the cantilever (6 pN), is about 58 nm, which is in good agreement with twice the length of a single PEG molecule, as expected.

**Figure 6.**
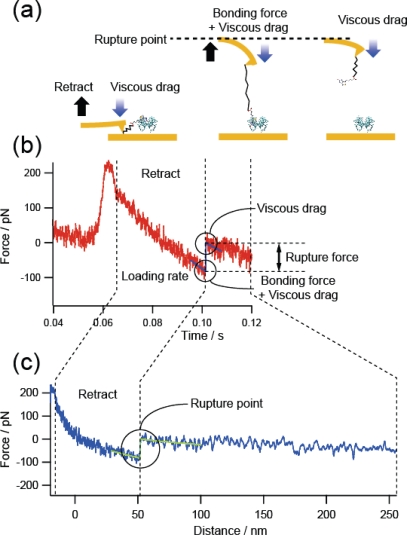
Schematic illustration of the force measurement and a typical force curve obtained at a high loading rate of 10^4^ pN/s to analyze the effect of the viscous drag on the cantilever.

Next, we examined the effect of the viscous drag on the cantilever. Figure 6 shows a force curve obtained at a high loading rate of 10^4^ pN/s for the B-PEG condition. The observed force includes the viscous drag on the cantilever. In fact, although the cantilever (Bio-lever) we used was smaller than the cantilever typically used for AFM, an increase in force was observed after the rupture event, where the retraction of the cantilever was accelerated to realize a constant loading rate without the formation of a molecular bond. To remove such undesirable effects, we estimated the rupture force by subtracting the force immediately after the rupture event. We examined the validity of this treatment by plotting 23 histograms obtained using three cantilevers with different spring constants and shapes (6 pN/nm, rectangular, Au-coated; 30 pN/nm, rectangular, Au-coated; and 20 pN/nm, triangular, noncoated Si_2_N_3_). No marked dependence on the type of cantilever was observed [15].

**Figure 7.**
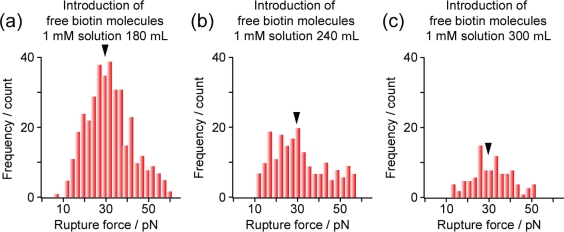
Change in binding rate caused by the introduction of free biotin molecules. The loading rate was set at 2.25 × 10^2^ pN/s for all measurements. In the measurements, the ratios of the number of detected streptavidin-biotin bonds to the total number of measurements were (**a**) 8.1%, (**b**) 4.5% and (**c**) 2.2%.

Thirdly, we confirmed the formation of a streptavidin-biotin structure from the reduction in bonding probability following the introduction of free biotin molecules into the solution. Figure 7 shows the change in binding rate, obtained under the SM condition, induced by the introduction of free biotin molecules. When free biotin molecules were added, the peak signal, indicated by an arrow, gradually decreased in intensity and eventually almost disappeared, whereas the peak position did not shift, as expected (Figure 7). The histograms in Figure 7 appear to show distributions with different shapes. This is due to the reduction in the amount of data obtained resulting from the introduction of free biotin molecules, which greatly reduced the probability of forming the streptavidin-biotin structure. In fact, each histogram in Figure 7, for which a sufficient amount of data was obtained, has the expected shape. This shows the importance of collecting a sufficient amount of data. In addition, the phosphate buffer ensures that the pH remains constant even when biotin molecules are added.

From these results, the observed force curves are considered to originate from the biotin-streptavidin interaction.

### 3.2. Rupture Force Distribution and Potential Barrier Position

**Figure 8.**
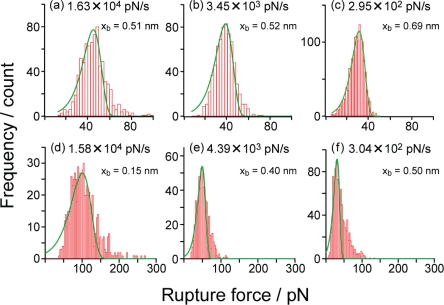
Typical histograms of the rupture forces obtained for the (**a–c**) SM and (**d–f**) B-PEG conditions in PBS (pH 7.4) at various loading rates.

Figure 8(a)–(c) shows typical histograms of the rupture forces obtained for the SM condition in PBS (pH 7.4) at the loading rates of (a) 1.63 × 10^4^ pN/s, (b) 3.45 × 10^3^ pN/s and (c) 2.95 × 10^2^ pN/s, where the modal rupture forces are (a) 45.1 pN, (b) 38.5 pN and (c) 31.0 pN. The modal rupture force decreases with the loading rate, as expected [7–26]. As mentioned in Section 2, the potential barrier position can be estimated by analyzing the shape of the rupture force distribution [15,16]. The solid green lines in Figure 8 show theoretical fitting curves (Equation 1), and the potential barrier position *x*_b_ obtained from each distribution in Figure 8 using the method in [15] and [16] is determined to be (a) 0.51 nm, (b) 0.52 nm and (c) 0.69 nm. The barrier position obtained by this method is estimated to be smaller than the actual value, when there is a broadening of the histogram. The results are consistent with the barrier position of 0.68 nm obtained from the slope in Figure 8, which is the best-fit value, because of the many data points used for estimation. The analysis based on the Bell model [9–26] is valid when the change in the potential shape of the system is small [9–26], and the obtained results validate the results of measurements performed in this study and indicate the stability of the potential barrier determined in each experiment.

On the other hand, Figure 8(d)–(f) shows typical histograms of the rupture forces obtained for the B-PEG condition in PBS (pH 7.4). Similarly to the results obtained for the SM condition, the modal rupture force decreases with the loading rate. From the theoretical fitting curves, the potential barrier position *x*_b_ is estimated to be (d) 0.15 nm, (e) 0.40 nm and (f) 0.50 nm. Although these estimated potential barrier positions are similar to those for the SM condition, the potential barrier position obtained from the fitting in Figure 8(d) is smaller than those obtained from the fitting in Figure 8(e),(f).

**Figure 9.**
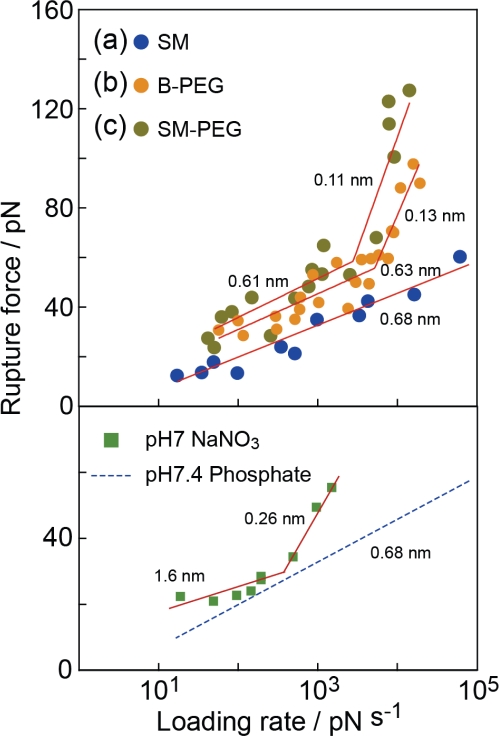
Relationships between the modal rupture force and the logarithm of the loading rate obtained for (**a**) SM (Figure 2(b)), (**b**) B-PEG [Figure 2(c)] and (**c**) SM-PEG (Figure 2(d)) conditions in 0.01 M PBS (pH 7.4). The barrier positions obtained from these results are shown in the figure. A similar experimental result obtained in 0.05 M sodium nitrate solution under the SM condition is also shown. The result obtained for the 0.01 M PBS (pH 7.4) solution shown in (b) is also indicated for comparison (blue dashed line).

When the Bell model can be applied with the assumption of the shape of potential landscapes described in previous studies [9,10,25], as in the present case, the deformation of the histogram is considered to be small at high and low loading rates. However, the shape of the histogram is broadened at a loading rate where two potential barriers are probed, as shown in Figure 8(d). In fact, two potential barriers are observed as will be discussed later for the results shown in Figure 9. There are other methods for the analysis of more complex cases [23,24].

### 3.3. Analysis of Molecular Interactions–Potential Barrier Position

Figure 9 shows the relationships between the modal rupture force and the logarithm of the loading rate obtained in 0.01 M PBS (pH 7.4) for the (a) SM, (b) B-PEG and (c) SM-PEG conditions shown in Figure 2. According to the DFS theory (Equation 2), each slope provides the potential barrier of one unbinding process, and the distance of the barrier position from the potential bottom, *x*_b_, can be deduced from the reciprocal of the gradient [7–26].

For the B-PEG and SM-PEG conditions, the gradient of the slopes exhibits a marked increase at 2 × 10^3^ pN/s. The potential barrier positions were estimated from the two slopes to be 0.13 ± 0.01 nm and 0.63 ± 0.09 nm for the B-PEG condition and 0.11 ± 0.05 nm and 0.61 ± 0.27 nm for the SM-PEG condition, In pioneering studies on the DFS of streptavidin-biotin complex, a potential landscape with two barrier widths (0.12 nm and 0.5 nm) was observed [9,10]. On the basis of the results obtained by X-ray diffraction and MD calculations, the small (0.12 nm) and large (0.5 nm) potential barrier positions were attributed to the direct hydrogen bonding of the inner amino acid residues, such as ASP128, TYR43 and ASN23, with the biotin molecule, and the indirect interaction of the middle sites, such as SER45, with the biotin molecule via water molecules, respectively [29–31]. The results obtained under B-PEG and SM-PEG conditions are consistent with that obtained in previous studies.

For the SM condition, in contrast, only one slope was observed and the potential barrier position estimated from this slope was 0.68 ± 0.05 nm. Namely, only the bridged bonding at the middle reaction sites was observed. To confirm the validity of this result, we plotted the values from several histograms obtained using three cantilevers with different spring constants and shapes (6 pN/nm, rectangular, Au-coated; 30 pN/nm, rectangular, Au-coated; and 20 pN/nm, triangular, noncoated Si_2_N_3_). No marked dependence on the type of cantilever was observed. Since the waiting time (30 ms or 300 ms), *i.e.*, the time before the cantilever was retracted after coming into contact with the sample, had no significant effect on the results, the bonds are considered to be fully formed [15].

**Figure 10.**
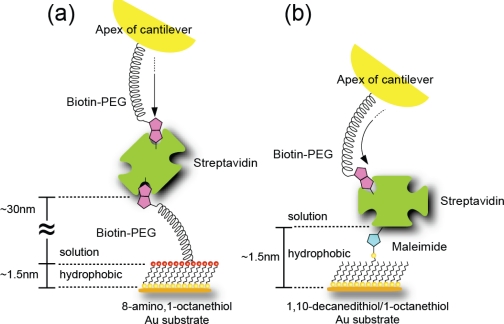
Schematic illustration of the site-selective analysis using two types of cross-linker. **(a)** B-PEG condition and **(b)** SM condition.

Although a more detailed study is necessary to determine the mechanism, it is considered that the biotin molecule attached to the cantilever can enter deep into the binding pocket and conjugate even with the inner amino acid residues in streptavidin under the B-PEG or SM-PEG condition (Figure 10(a)). Under the SM condition (Figure 10(b)), in contrast, this process is prevented. This may be due to the lack of flexibility or to the effect of the hydrophobicity of the substrate surface on the formation of a hydrogen bond. Since the SAM of octanethiol molecules formed on the Au substrate has a hydrophobic characteristic, the reduction in the distance between the streptavidin molecule and the substrate may increase the effect of the hydrophobicity of the substrate. The potential barriers obtained for the 3 nm linkers in [12] (in Table 1) are similar to those obtained for the B-PEG condition, suggesting that the effect of the molecular chain is negligible in the case of 3 nm linkers, enabling precise measurements. Since the linker length for the SM condition is 1.5 nm, the effect of the SM condition may occur between linker lengths of 1 nm and 3 nm.

With this method, bonding at the middle reaction sites can be selectively probed. To analyze the origins of the potential barriers of 0.68 nm and 0.63 nm in more detail, we changed the buffer solution from phosphate (pH 7.4) to sodium nitrate (pH 7) [15]. The measurements were performed under the SM condition. For the sodium nitrate, the slope changed at a loading rate of about 10^2^–10^3^ pN/s, and the two potential barrier positions were estimated to be 0.26 nm and 1.6 nm.

Since no bond corresponding to the potential barriers of 0.68 nm and 0.63 nm exists in the 0.05 M sodium nitrate solution, the bond related to the potential barrier positions of 0.68 nm and 0.63 nm is suggested to be formed by molecular bridging between the amino acid residues at the middle reaction sites and the biotin molecule [15]. Namely, the potential barrier positions of 0.68 nm and 0.63 nm are formed by the phosphate molecules in the buffer solution [15] instead of the water molecules, in contrast to the mechanism predicted in previous studies [9,10].

The bonding at the inner or middle sites was distinguished and separately analyzed for the SM, B-PEG and SM-PEG conditions. To the best of our knowledge, this is the first demonstration of site-selective analysis by DFS. Using a combination of cross-linkers and the atomic force microscope that we developed for precise analysis by DFS, direct and bridging interactions at each reaction site in a ligand-receptor system were distinguished and individually analyzed.

### 3.4. Analysis of Molecular Interactions–Lifetime

The lifetime of bonds can be estimated from the intercept obtained by extrapolating the linear relationship between the modal rupture force and the logarithm of loading rate (Equation 2), as shown in Figure 9 [7–26]. The rupture forces obtained for the B-PEG condition were larger than those for the SM condition at all loading rates, as shown in Figure 9, and the lifetimes obtained for the SM, B-PEG and SM-PEG conditions were 1.0 s, 6.4 s and 13 s, respectively.

**Figure 11.**
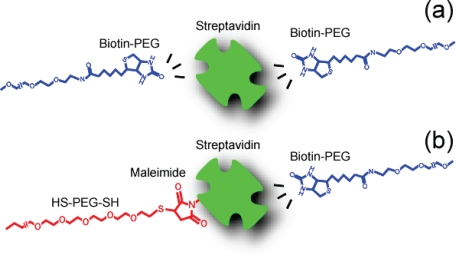
Schematic illustrations of the rupture types for (**a**) B-PEG and (**b**) SM-PEG conditions.

Since two rupture points exist for the B-PEG condition (Figure 11(a)) and one rupture point exists for the SM-PEG condition (Figure 11(b)), the rupture probability for the B-PEG condition is considered to be two times larger than that for the SM-PEG condition. Namely, the lifetime for the B-PEG condition is expected to be half that for the SM-PEG condition. From the result of the B-PEG (PEG-biotin-streptavidin-biotin-PEG) condition (6.4 s), the lifetime for streptavidin-biotin-PEG is estimated to be 12.8 s [12]. This value is in good agreement with the lifetime obtained for the SM-PEG condition (13 s). The obtained values are ten times larger than that obtained for the SM condition. This is caused by the fact that the direct bonds with the inner amino acid residues, such as ASP128, TYR43 and ASN23, are stable under the B-PEG and SM-PEG conditions because the effect of the hydrophobicity of the substrate, for example, is less than that under the SM condition. Similarly to the potential barriers mentioned in Section 3.3, the lifetime obtained for the 3 nm linkers in the previous experiment [12] (in Table 1) is similar to that obtained for the B-PEG condition, suggesting that the effect of the SM condition is negligible at 3 nm from the substrate in the case of steric hindrance.

The lifetimes obtained from DFS measurements are, in general, much shorter than that expected from the dissociation constant (*K*_d_ ∼ 10^−15^) of the streptavidin-biotin complex [28,29]. This is because the dissociation constant is measured under an equilibrium condition where biotin molecules form bonds with all three group sites in streptavidin. However, the lifetime of each bond is evaluated using the results obtained by DFS measurement instead of the total strength, resulting in the observed shortening of the lifetime.

The lifetime, and thereby the observed rupture force, changes with the experimental conditions. Despite this fact, different conditions were used in previous experiments. Namely, some data were obtained using a cantilever without a PEG molecule, and other data were obtained using a PEG molecule at different buffer concentrations. In addition, the loading rate was not kept constant. Therefore, the difference in lifetime between our study and previous studies (for example, that indicated in [9] may be caused by the differences in pH and ion concentration. Such variations among the reported values must be due to important effects yet to be understood in detail, which, therefore, must be clarified in the future. In any case, a precise DFS measurement technique will be essential for the analysis.

### 3.5. Analysis of Molecular Interactions – Reaction Processes

The results obtained in this study are shown in Table 2. As shown in Figure 1, the binding pocket of streptavidin has several reaction sites with a hydrogen-bonding network, which are classified into the following three groups depending on the distance from the bottom of the binding pocket: (1) the inner binding sites of amino acid residues SER27, ASN23, TYR43 and ASP128, (2) the middle binding sites SER45 and THR90, and (3) the outer binding sites ASN49 and SER88 [28,29]. The barrier positions observed correspond to these three types: two direct bonds at inner and middle sites, and one indirect bond via solvent molecules at middle sites. Since the –COOH of the biotin molecule was attached to the PEG molecule, the interactions between the –COOH of the biotin molecule and the outer binding sites ASN 49 and SER88 are considered to be very weak, resulting in the absence of potential barriers between a biotin-PEG molecule and the outer binding sites.

**Figure 12.**
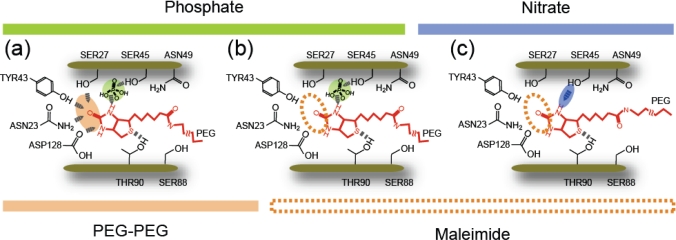
Schematics of the bonding structures of streptavidin-biotin complex.

These results obtained by site-selective analysis enable us to discuss the step-by-step processes for various conditions: For the B-PEG and SM-PEG conditions in PBS, the biotin molecule is trapped at the inner sites due to direct bonding and at the middle sites via the bridging of buffer molecules (Figure 12(a)). For the SM condition in PBS, the biotin molecule is not trapped at the inner sites but only at the middle sites via the bridging of buffer molecules (Figure 12(b)). For the SM condition in sodium nitrate solution, the biotin molecule is trapped at the middle sites via the hydrogen bonding of amino acid residues (Figure 12(c)).

## 4. Conclusions

We have developed an atomic force microscopy technique that enables precise analysis of the molecular interactions on the basis of DFS. The landscapes of streptavidin-biotin interactions obtained by this technique are consistent with theoretical predictions, indicating the importance of the precise measurement conditions realized by this microscopy technique. Lifetimes were also well analyzed. Furthermore, using a combination of cross-linkers and the atomic force microscope that we developed, site-selective measurement was carried out. Direct and bridging interactions at each reaction site in a typical ligand-receptor system, *i.e.*, the streptavidin-biotin complex, were clearly distinguished and individually analyzed. This methodology will provide a foundation for further advances in biophysics and chemistry and their applications, such as designing and controlling the mechanism of chemical reactions between functional molecules.

## Figures and Tables

**Table 1.** Bell model parameters obtained from previous studies: potential barrier positions (*x*_b_) and dissociation rates (*k*).

**Method**	**Surface spacer molecule and length**	**Tip cross-linker and length**	**Solution**	**Potential barrier*x***_**b1**_**/nm**	**Potential barrier*x***_**b2**_**/nm**	***k***_**1**_**/s^−1^**	***k***_**2**_**/s^−1^**	**Reference**
**BFP**	PEG (30 nm)	PEG (30 nm)	PBS (pH 6.8)	0.5	0.12	6.14 ×10^−5^	2.9	[9]
**AFM**	Agarose polymer (-)	BSA (3 nm∼)	PBS (pH 7.2)	0.49	0.05	1.67 ×10^−5^	2.09	[11]
**AFM**	BSA (3 nm∼)	BSA (3 nm∼)	-	0.6	0.14	0.2	54	[12]
**AFM**	poly-L-lysine (-)	Glutaraldehyde (1 nm)	PBS (pH 7.2)	0.081	0.024	0.56	2.98	[13]

**Table 2.** Potential barrier positions obtained for various conditions.

**pH**	**condition**	**Buffer solution**	**Potential barrier position (x**_**b**_**)**			
pH7.4	SM	Phosphate	-	-	0.68 nm	-
pH7	SM[15]	NaNO_3_	-	0.26 nm	-	1.6 nm
pH7.4	B-PEG [15]	Phosphate	0.13 nm	-	0.63 nm	-
pH7.4	SM-PEG	Phosphate	0.11 nm	-	0.61 nm	-
pH6.8	R. Merkel *et al*.[9,10]	Phosphate	0.12 nm	-	0.50 nm	-

Bond type			Direct bond	Direct bond	Salt bridge	-

Amino acid residue			ASP128, TYR43, ASN23	SER27, SER45	SER27, SER45	-

Barrier positions by MD[ 31]			0.10 nm	0.26 nm	-	-

Reaction area			inner	middle	-	-
